# Supplementary material for: Primary care screening for sexually transmitted infections in the United States from 2019 to 2021
Source: PLoS One. 2025 Jun 2;20(6):e0325097. doi: 10.1371/journal.pone.0325097 (PMC12129226; doi:10.1371/journal.pone.0325097)
Supplement: S1 Table — ICD, International Classification of Diseases; CPT, Current Procedural Terminology; HCPCS, Healthcare Common Procedure Coding System. LOINC: Logical Observation Identifiers, Names and Codes. (PDF) [file pone.0325097.s001.pdf]

S1 Table. Procedure codes used to identify the tests for chlamydia, gonorrhea, syphilis, and HIV. ICD, International Classification of Diseases; CPT, Current Procedural Terminology; HCPCS, Healthcare Common Procedure Coding System. LOINC: Logical Observation Identifiers, Names and Codes.

	ICD/CPT/HCPC	LOINC	LabCorp/Quest Diagnostic Test Codes	Other
Chlamydia	86631, 86632, 87110, 87270, 87320, 87490, 87491, 87810, 87800, 87492, 87801	14463-4, 43304-5, 21190-4, 45076-7, 16600-9, 50387-0, 6357-8, 43404-3, 45084-1, 42931-6, 21613-5, 0257-6, 64017-7, 53926-2, 57288-3, 21613-5	183194, 11363, 70051, 16506, 188078, 183616, 183160, 188698, 188672, 186134, 186098, 183161, 186114, 188610	LAB202, LAB7080, LAB2728, LAB3985, CHPCR, LAB1376, 70043800
Gonorrhea	87590, 87591, 87580, 87800, 87592, 87801, 87850	60256-5, 43305-2, 5028-6, 21416-3, 47387-6, 43403-5, 50388-8, 0257-6, 24111-7, 6490-7, 32705-6, 60255-7, 57289-1, 64017-7	11363, 70051, 16506, 183616, 183194, 183160, 188698, 188672, 188730, 188748, 180104, 188086, 188632, 188634, 186122, 186135, 186106, 183618	LAB202, LAB7080, GCPCR, LAB3985, LAB2727, LAB1376, 70043900, 6021, 54021, 53971
Syphilis	86592, 86593, 86780, 0065U, 0064U	47236-5, 6561-5, 47238-1	90349, 082345, 012005, 006379	
HIV	80081, 86689, 86701, 86702, 86703, 87391, 87537, 87389, 87390, 87534, 87535, 87536, 87538, 87539, G0432, G0433, G0435, G0475, Z11.4, S3645	29893-5, 56888-1, 7917-8, 7918-6, 14092-1, 16975-5, 21007-0, 24012-7, 29327-4, 30361-0, 31201-7, 32571-2, 33806-1, 33807-9, 33866-5, 34591-8, 34592-6, 35437-3, 35439-9, 40732-0, 40733-8, 41144-7, 41145-4, 43009-0, 43011-6, 43012-4, 43013-2, 44531-2, 44532-0, 44533-8, 44872-0, 48345-3, 5221-7, 5222-5, 5225-8, 54086-4, 57975-5, 68961-2, 69668-2, 73905-2, 73906-0, 75666-8, 77685-6, 7919-4, 80203-3, 80387-4,	083935, 91431, 12075	

		81641-3, 83101-6, 85037-0, 85686-4, 86233-4, 89365-1, 95523-7, 95524-5, 97860-1, 97861-9		
