# Supplementary material for: Primary care screening for sexually transmitted infections in the United States from 2019 to 2021
Source: PLoS One. 2025 Jun 2;20(6):e0325097. doi: 10.1371/journal.pone.0325097 (PMC12129226; doi:10.1371/journal.pone.0325097)
Supplement: S2 Table — (PDF) [file pone.0325097.s002.pdf]

S2 Table. Annual number of tests per 100,000 patients and the 95% confidence intervals (CIs) for the chlamydia, gonorrhea, syphilis, and HIV in the American Family Cohort from 2019 to 2021.

	Chlamydia, test counts per 100,000 (95% CI)	Gonorrhea, test counts per 100,000 (95% CI)	Syphilis, test counts per 100,000 (95% CI)	HIV, test counts per 100,000 (95% CI)
All patients				
2019	3,593 (3,572-3,613)	2,129 (2,110-2,147)	1,702 (1,686-1,718)	1,860 (1,844-1,876)
2020	2,355 (2,337-2,373)	2,207 (2,187-2,226)	1,641 (1,624-1,657)	1,756 (1,740-1,772)
2021	2,181 (2,162-2,200)	2,057 (2,036-2,078)	1,474 (1,457-1,491)	1,723 (1,706-1,741)
Female				
2019	4,666 (4,635-4,697)	2,488 (2,462-2,513)	1,897 (1,875-1,919)	2,024 (2,002-2,045)
2020	3,066 (3,040-3,093)	2,736 (2,707-2,765)	1,936 (1,912-1,959)	1,970 (1,948-1,992)
2021	2,732 (2,704-2,760)	2,434 (2,404-2,464)	1,626 (1,603-1,650)	1,816 (1,792-1,840)
Male				
2019	2,026 (2,003-2,050)	1,592 (1,567-1,616)	1,383 (1,361-1,405)	1,582 (1,560-1,604)
2020	1,315 (1,294-1,335)	1,406 (1,382-1,431)	1,174 (1,152-1,195)	1,408 (1,386-1,430)
2021	1,383 (1,359-1,406)	1,489 (1,461-1,517)	1,220 (1,196-1,244)	1,555 (1,530-1,581)
Non-Hispanic Black or African American				
2019	10,501 (10,362-10,639)	5,300 (5,189-5,411)	5,306 (5,191-5,420)	4,736 (4,641-4,832)
2020	7,832 (7,706-7,959)	6,802 (6,669-6,936)	5,855 (5,730-5,981)	5,246 (5,140-5,351)
2021	7,327 (7,191-7,464)	6,820 (6,670-6,970)	5,076 (4,951-5,200)	4,898 (4,786-5,010)
Hispanic or Latino				
2019	4,980 (4,905-5,054)	3,611 (3,542-3,679)	2,442 (2,387-2,497)	2,819 (2,762-2,876)
2020	3,053 (2,991-3,114)	2,909 (2,844-2,973)	2,088 (2,034-2,142)	2,459 (2,403-2,515)
2021	3,134 (3,062-3,205)	2,969 (2,893-3,044)	2,106 (2,045-2,166)	2,905 (2,836-2,974)
Non-Hispanic Whites				

2019	2,822 (2,798-2,845)	1,482 (1,463-1,501)	1,166 (1,149-1,183)	1,319 (1,303-1,336)
2020	1,697 (1,678-1,716)	1,500 (1,479-1,520)	1,100 (1,083-1,117)	1,161 (1,144-1,178)
2021	1,445 (1,426-1,465)	1,274 (1,252-1,295)	863 (847-880)	1,014 (997-1,031)
