# Supplementary figures and images for: Primary care screening for sexually transmitted infections in the United States from 2019 to 2021
Source: PLoS One. 2025 Jun 2;20(6):e0325097. doi: 10.1371/journal.pone.0325097 (PMC12129226; doi:10.1371/journal.pone.0325097)

**S1 Fig.** Distribution of the 753 primary care practices by U.S. states.

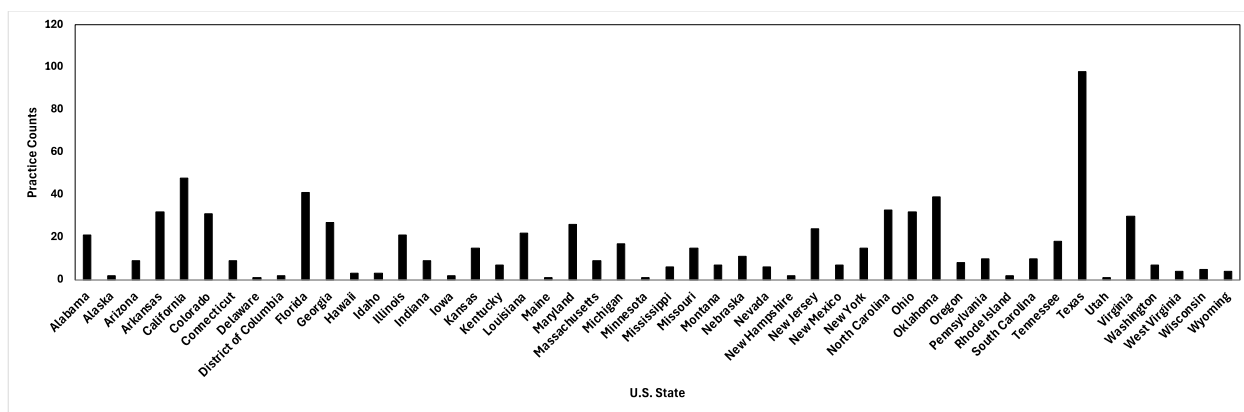

Supplement: S1 Fig — (PDF) [file pone.0325097.s003.pdf]
