# Supplementary material for: Primary care screening for sexually transmitted infections in the United States from 2019 to 2021
Source: PLoS One. 2025 Jun 2;20(6):e0325097. doi: 10.1371/journal.pone.0325097 (PMC12129226; doi:10.1371/journal.pone.0325097)

S2 Fig. Monthly number of tests per 100,000 patients for chlamydia, gonorrhea, syphilis, and HIV in the American Family Cohort from 2019 to 2021 by gender, age standardized to the U.S. standard population. Primary care visits from seven women-specific practices excluded. The vertical dashed line represents the beginning of the stay-at-home order in March 2020.

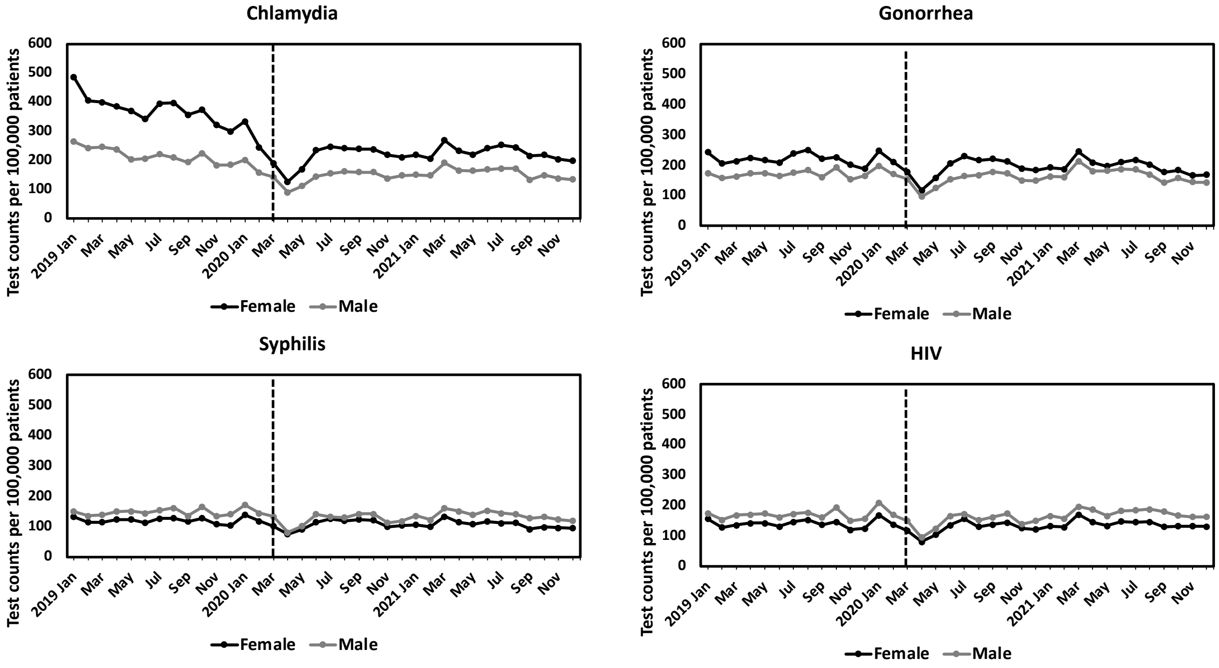

Supplement: S2 Fig — Primary care visits from seven women-specific practices excluded. The vertical dashed line represents the beginning of the stay-at-home order in March 2020. (PDF) [file pone.0325097.s004.pdf]
