# Supplementary material for: Primary care screening for sexually transmitted infections in the United States from 2019 to 2021
Source: PLoS One. 2025 Jun 2;20(6):e0325097. doi: 10.1371/journal.pone.0325097 (PMC12129226; doi:10.1371/journal.pone.0325097)
Supplement: Attachment — Submitted filename: Response letter STI Y2_2024_6.docx [file pone.0325097.s006.docx]

May 29, 2024

Janet E Rosenbaum, Ph.D.

Academic Editor

PLOS ONE

Dear Dr. Rosenbaum:

Thank you for the opportunity to revise and resubmit our article, “Primary care usage patterns associated with sexually transmitted infections during COVID-19” (**ID: PONE-D-24-07258**) for consideration in PLOS ONE.

As requested, we have incorporated the edits suggested by the editor and the two reviewers, as itemized below and as marked in the attached files. We have attached a version of a mark-up copy of the manuscript in ‘tracked changes’ as well as an unmarked version. We have also uploaded a cover letter to include the updated competing interests statement and financial disclosure.

Thank you for your consideration.

Best regards,

Shiying Hao

Journal requirements:

When submitting your revision, we need you to address these additional requirements.

1. Please ensure that your manuscript meets PLOS ONE's style requirements, including those for file naming. The PLOS ONE style templates can be found at

<https://journals.plos.org/plosone/s/file?id=wjVg/PLOSOne_formatting_sample_main_body.pdf> and

<https://journals.plos.org/plosone/s/file?id=ba62/PLOSOne_formatting_sample_title_authors_affiliations.pdf>

**Response: Thanks for the guidance. We’ve updated our manuscript to meet the style requirements.**

2. Thank you for stating the following financial disclosure:

“Funder: Centers for Disease Control and Prevention (CDC)

Award Number: 75D30122P12974

Grant Recipient: American Board of Family Medicine, Inc.

URL: [https://www.cdc.gov”](https://www.cdc.xn--gov-9o0a)

Please state what role the funders took in the study.  If the funders had no role, please state: "The funders had no role in study design, data collection and analysis, decision to publish, or preparation of the manuscript."

If this statement is not correct you must amend it as needed.

Please include this amended Role of Funder statement in your cover letter; we will change the online submission form on your behalf.

**Response: We have included this statement in the updated cover letter.**

3. Thank you for stating the following in the Competing Interests section:

“Neil Kamdar serves as a consultant at the University of North Carolina, Chapel Hill, Sheps Center for Health Policy, and the University of New Mexico, Department of Emergency Medicine. Other authors have no conflicting or competing interests.”

Please confirm that this does not alter your adherence to all PLOS ONE policies on sharing data and materials, by including the following statement: "This does not alter our adherence to  PLOS ONE policies on sharing data and materials.” (as detailed online in our guide for authors <http://journals.plos.org/plosone/s/competing-interests>).  If there are restrictions on sharing of data and/or materials, please state these. Please note that we cannot proceed with consideration of your article until this information has been declared.

Please include your updated Competing Interests statement in your cover letter; we will change the online submission form on your behalf.

**Response: We have included this statement in the updated cover letter.**

4. We note that you have indicated that there are restrictions to data sharing for this study. PLOS only allows data to be available upon request if there are legal or ethical restrictions on sharing data publicly. For more information on unacceptable data access restrictions, please see <http://journals.plos.org/plosone/s/data-availability#loc-unacceptable-data-access-restrictions>.

Before we proceed with your manuscript, please address the following prompts:

a) If there are ethical or legal restrictions on sharing a de-identified data set, please explain them in detail (e.g., data contain potentially identifying or sensitive patient information, data are owned by a third-party organization, etc.) and who has imposed them (e.g., a Research Ethics Committee or Institutional Review Board, etc.). Please also provide contact information for a data access committee, ethics committee, or other institutional body to which data requests may be sent.

b) If there are no restrictions, please upload the minimal anonymized data set necessary to replicate your study findings to a stable, public repository and provide us with the relevant URLs, DOIs, or accession numbers. For a list of recommended repositories, please see

<https://journals.plos.org/plosone/s/recommended-repositories>. You also have the option of uploading the data as Supporting Information files, but we would recommend depositing data directly to a data repository if possible.

We will update your Data Availability statement on your behalf to reflect the information you provide.

**Response: We cannot make the data publicly available due to contractual limitations and the inclusion of sensitive information and PHI. However, individuals wishing to access the data may contact the Stanford Center for Population Health Sciences at phsdatacore@stanford.edu. Upon completion of security, regulatory and contractual requirements, individuals may access the data.**

5. We notice that your supplementary figures and tables are included in the manuscript file. Please remove them and upload them with the file type 'Supporting Information'. Please ensure that each Supporting Information file has a legend listed in the manuscript after the references list.

**Response: We have uploaded each appendix as a separate supporting information file. A legend list was added to the manuscript.**

Reviewer #1: My concerns include the following:
1. Chlamydia (CT) and gonorrhea (GC) can be (and should be) done with swab specimen or urine, so it's not clear why the GC tests were significantly greater than the CT tests. A provider may only order one of the tests but to have such a big difference makes me question the ordering process.

**Response: We thank the reviewer for raising these concerns. While we recognize that there exist differences in the chlamydia and gonorrhea testing rates, the database does not provide insights specifically into practice pattern differences in the ordering of tests. Primary care providers’ data are documented from each practice’s electronic health records but does not provide information regarding the process regarding how tests are ordered. We have documented in the manuscript regarding this data limitation, and have added some language into the limitations section of the Discussion. It is important to recognize that future work may require evaluation of documentation standards regarding testing and/or STI co-testing for chlamydia and gonorrhea alongside the clinical decision making and ordering process.**

2. CT and GC can be in the vagina/cervix, penis, throat, and/or rectum. It's possible that one patient had a syphilis test, an HIV test, a throat CT/GC test, a rectal CT/GC test, and a urine CT/GC test at the same visit. It's not clear in the manuscript if this was accounted for (extragenital site vs genital site CT/GC tests). And for the positive rates, would a person with a positive GC result of the throat and the rectum on the same day we counted as 1 positive GC or 2 positive GC tests?

**Response: We thank the reviewer for highlighting these. We have revised the “Outcome definitions” section to clarify: “A chlamydia or gonorrhea test could be a test from genital, throat, or rectal areas. A person with positive chlamydia or gonorrhea results of genital, throat, or rectal areas on the same day were counted as one positive chlamydia or gonorrhea test.”**

3. Why were syphilis and HIV positivity rates not done, and not mentioned in the abstract results? Syphilis is complicated I understand since a positive/reactive test result doesn't necessarily mean an untreated or "new" infection.

**Response: We thank the reviewer for raising these concerns. Unlike chlamydia and gonorrhea, it is hard to define a “new” incidence for syphilis and HIV with our data. A presumptive diagnosis of syphilis requires use of two laboratory serologic tests, so one positive test result of syphilis doesn’t necessarily mean a new infection. As for HIV, since it is a lifetime condition and some patients may have already been with it before entering the PRIME Registry, we are unable to determine a new incident HIV simply based on positive test results. This is a limitation of our data source. Given the complexity and uncertainty of calculating the positivity rates for syphilis and HIV, we didn’t include positivity rates for these two conditions in the manuscript.**

4. What were the outcomes of the age groups? It would be informative to know the p-value of age group and testing outcomes. Adolescents and young adults have the highest rates of bacterial STIs but were they more or less likely to be tested during COVID?

**Response: We agree with the reviewer that it would be informative to know the p-value of age group and testing outcomes. We’ve added the following findings in the Results.**

**For all four types of STI testing (chlamydia, gonorrhea, syphilis, and HIV), patients aged 10-39 had higher probabilities (P<0.001, Chi-squared test) of being tested from 2019 to 2021 than other age groups.**

**During the COVID, patients aged 10-24 (i.e. adolescents and young adults) consistently had higher testing rates (P<0.001, Chi-squared test) than others (chlamydia: 4% vs 1% in 2020 and 4% vs 0.9% in 2021; gonorrhea: 4% vs 1% in 2020 and 4% vs 0.9% in 2021; syphilis: 2% vs 0.8% in 2020 and 2% vs 0.7% in 2021; HIV: 3% vs 1% in both 2020 and 2021).**

5. Department of Health STI clinics are a common place for people to go for STI testing since it is a public clinic (and testing and treatment is free if not insured) along with family planning clinics - neither of these types of clinics were mentioned on page 5-6 when listing U.S. STI testing site types.

**Response: We agree with the reviewer that STI clinics and family planning clinics are common places for STI testing and care. We have added these to the U.S. STI testing site types in the manuscript: “Many healthcare settings are utilized for STI testing in the U.S., including *STI clinics, family planning clinics*, hospitals, emergency departments, urgent care centers, and physician offices, and differences in how STIs are managed in these different locations have been noted.”**

6. Several typos: Human Immunodeficiency Virus should be human immunodeficiency virus on page 5; on abbreviations, you have STI and STIs - I would just use STIs or remove the "s" on infections in the STI write out; Reference #2 has a broken link; page 8, "sexr" should be "sex".

**Response: We thank the reviewer for identifying the typos. We have fixed typos on page 5 and page 8. We have removed “STIs-Sexually transmitted infections” from the list of abbreviations. The link in Reference #2 has been fixed.**

7. Gay, bisexual, and other MSM may be the reason more "men" were tested for HIV and syphilis compared to women. However, during this time period, and worse now, congenital syphilis rates were increasing, so women should be being tested along with heterosexual men, but none of this is mentioned. I'm assuming there is no sexual orientation identity demographic data in the data set used, but this should be listed as a limitation.

**Response: We agree with the reviewer that lack of sexual orientation is a limitation for our study. We mentioned this as a study limitation: “Incomplete demographic data was also a limitation. Sexual orientation and gender diversity information was not available in our data.”**

Reviewer #2: Reviewer comments
Thank you for considering me as a reviewer for the study entitled: “Primary care usage patterns associated with sexually transmitted infections during COVID-19” which I read with great interest. The authors examined trends in STI testing and positivity before and during the COVID-19 pandemic in the primary care setting using retrospective data from the PRIME Registry collected from January 1, 2019-December 31, 2021.
Several questions remain that need further clarification please see below.

**Response: Thank you for recognizing the contribution of this manuscript. We have revised the manuscript throughout to be more concise and precise, and detail these revisions below.**

Abstract
Methods
“We calculated age-standardized monthly and annual STI testing rates and stratified by gender and race and ethnicity.”
Reviewer: I think it would help to clarify which STI you examined.

**Response: We thank the reviewer for highlighting this. We’ve added STI categories into the sentence.**

“We calculated age-standardized monthly and annual STI testing rates and stratified by sex and race and ethnicity. We also generated quarterly and annual rates for test positivity.”
Reviewer: What was the rational to calculate monthly and annual STI testing rates but for test positivity quarterly and annual rates?

**Response: We thank the reviewer for raising these concerns. It was due to the small number of tests that were qualified for the test positivity calculation in our analysis. The small number could restrict us from getting a sense of trend, so we only calculate quarterly and annual rates for test positivity.**

Introduction
Reviewer: I think it would be good for the understanding of the results to include information on times of lockdowns in the US.

**Response: We added to the Introduction: “Stay-at-home orders were issued in March or April 2020 in the U.S.”**

Methods
Reviewer: Based on what did you identify positive test results, how are they coded in the AFC EHR? How likely is miscoding? In S2 Appendix you provided the codes to identify tests, I think it would be good to provide the codes used to identify positive tests. Using for example ICD-10 codes for HIV can be tricky due to miscoding.

**Response: AFC EHR has a separate table that includes the results of observations generated by laboratories, imaging procedures, and other procedures. Laboratory results are typically generated by laboratories providing analytic services. These observations are based on analysis of specimens obtained from the patients and submitted to the laboratory. In this table, a typical record includes a patient id, an observation code (in forms of CPT, LOINC, or other lab test codes) and descriptions received from the practice, and the date and value of the result for this observation.**

**For STI tests, most of the result values were coded as “negative”, “not detected”, “detected”, or “positive”. We determined positive tests as those with result values coded as “detected” or “positive”. We added the following text to “Outcome definition”:**

**“The evidence of having a positive test was those having results coded as “positive” or “detected” in the EHR.”**

**We thank the reviewer for highlighting the concern about the miscoding in our data source. Data integrity and coding accuracy in real-world EHR systems could be essential parts that impact patient care. Literature indicated that EHR-related errors could be caused by poor design of the platform, incomplete documentation, and insufficient training on coding (PMID: 38293266; 24159271; 35646364). However, due to the unavailability of the original patients’ files and detailed laboratory test documentation in AFC EHR, we were unable to quantify the miscoding and its potential impact on our analysis. This is a limitation of our study.**

Reviewer: Did you distinguish between different stages of Syphilis and latent and acute Syphilis?

**Response: We didn’t collect ICD diagnosis codes for syphilis in this study, so we were unable to determine the stage of syphilis or distinguish different stages.**

“Patients with missing information for age were excluded.”
Reviewer: I assume this was because you used age for the standardization?

**Response: That’s correct. Age was the critical factor to implement the standardization process in our study, so we excluded patients with missing age information.**

“Our primary outcome was the monthly and annual rates for testing for chlamydia, gonorrhea, syphilis, and HIV during the study period, …”
“Our secondary outcome was the test positivity for chlamydia and gonorrhea, respectively.”
Reviewer: Why did you only look at chlamydia and gonorrhea for the secondary outcome?

**Response: We thank the reviewer for highlighting this. Unlike chlamydia and gonorrhea, it is harder to define a “new” incidence for syphilis and HIV using our data. A presumptive diagnosis of syphilis requires use of two laboratory serologic tests, so one positive test result of syphilis doesn’t necessarily mean a new infection. As for HIV, since it is a lifetime condition and some patients may have already been with it before entering the PRIME Registry, we are unable to determine a new incident HIV simply based on positive test results. This is a limitation of our data source. Given the complexity and uncertainty of calculating the positivity rates for syphilis and HIV, we didn’t include positivity rates for these two conditions in the manuscript.**

“Monthly and annual rates of STI tests were calculated and presented for all patients and by category (sex: female and male; race and ethnicity: non-Hispanic Whites, non-Hispanic Blacks or African Americans, Hispanic/Latino).”
Reviewer: Does the data only provide female or male sex, what about sex divers?

**Response: Nearly 99% of the patients were labeled as female, male, or unknown in our data. It doesn’t contain further information about sex diversity. We mentioned this as a study limitation: “Incomplete demographic data was also a limitation. Sexual orientation and gender diversity information was not available in our data.”**

Results
Reviewer: To avoid confusion I would reorder the sentences and already include the total number of tests in the first paragraph as follows:
A total of 753 primary care practices (S4 Appendix) and 4,410,609 patients were included in the study. Patients had a total of 233,510 tests for chlamydia, 135,559 tests for gonorrhea, 109,909 tests for syphilis, and 135,953 tests for HIV in 2019 – 2021. Among these patients, 180,558 had one or more STI tests (chlamydia: 132,076 patients; 702 practices; gonorrhea: 86,672 patients; 438 practices; syphilis: 69,336 patients; 475 practices; HIV: 90,083 patients; 552 practices) in 2019 – 2021.

**Response: We thank the reviewer for the suggestion. We’ve reordered the sentences accordingly.**

Trends of STI tests
Reviewer: I would think about including the information into a table as there are a lot of numbers and CI presented and a table would help to compare the different rates over time.

**Response: We agree with the reviewer that including a table of numbers and CIs of testing trends would be helpful. We’ve created a table (S3 Table) to show the monthly number of STI tests from 2019 through 2021.**

“Rates of all four STI tests further decreased since August 2021 (Fig1).”
Reviewer: See comment on lockdown times to better understand the results.

**Response: We agree with the reviewer that adding lockdown times would help to better understand the results. We've included several references (ref [30]-[34]) to the Discussion that illustrate the announcement or extension of the state of emergency due to new waves of COVID-19 cases in the U.S. during 2021. These could help to explain the reduction in STI testing rates that we observed from our data since August 2021.**

“The annual rates of chlamydia tests in 2020 (2,355 [95% CI: 2,337-2,373] per 100,000 patients) and 2021 (2,181 [95% CI: 2,162-2,200] per 100,000 patients) were lower than in 2019 (3,592 [95% CI: 3,572-3,613] per 100,000 patients), while the annual rate of gonorrhea tests in 2020 (2,207 [95% CI: 2,187-2,226] per 100,000 patients) was higher than in 2019 (2,129 [95% CI: 2,110-2,147] per 100,000 patients) (S5 Appendix).”
Reviewer: How is chlamydia and gonorrhea tested. Is it not tested together in a PCR test?

**Response: We thank the reviewer for raising these concerns. Our results show that the overall testing rate for chlamydia was higher than gonorrhea in 2019, and the two testing rates were getting closer in 2020 and 2021. While we recognize that there exist differences in the chlamydia and gonorrhea testing rates, the database does not provide insights specifically into practice pattern differences in the ordering of tests. Primary care providers’ data are documented from each practice’s electronic health records but does not provide information regarding the process regarding how tests are ordered. We have added some language into the limitations section of the Discussion.**

Table 1. Age in years
Reviewer: How do you explain the <10 years old with STI test in the data?

**Response: In our data, around 0.2% of the patients were less than 10 when they had STI tests. Reasons for testing in this age group included child sexual abuse or suspected exposure to sexually transmitted infections. Another possible reason could be data entry error, such as clinicians providing incorrect dates of birth.**

Reviewer: See comment before on why testing rates monthly and test positivity quarterly?

**Response: We thank the reviewer for raising these concerns. It was due to the small number of tests that were qualified for the test positivity calculation in our analysis. The small number could restrict us from getting a sense of trend, so we only calculate quarterly and annual rates for test positivity.**

Reviewer: Would it be possible to stratify test positivity also by age, sex, race and ethnicity or are numbers too small?

**Response: The numbers of tests for each STI qualified for test positivity calculation would be small after stratification, which might prevent meaningful statistical analysis.**

Reviewer: Fig 4. I would suggest to change the order of the legend accordingly to the order in the graph. Non-Hispanic White, Hispanic, Non-Hispanic Black/African American.

**Response: The figure legend was changed accordingly.**

“Relatedly, the increase in the case incidence as well as the various lockdowns during the pandemic period had led to a decrease in primary care practices remaining open or their overall capacity to manage the same patient volumes as they did during the prepandemic period.[25]”
Reviewer: See comment before. I´d suggest to provide background info on times of lockdown.

**Response: Following the reviewer’s suggestion, we've added to the Introduction the time of issuing the stay-at-home order in the U.S. in 2020, and included several references (ref [30]-[34]) to the Discussion that illustrate the announcement or extension of the state of emergency due to new waves of COVID-19 cases in the U.S. during 2021.**

“Following the guidance, syphilis and gonorrhea were made a top priority by health departments, resulting in the significant decline in chlamydia tests due to the limited resources and the increase in gonorrhea tests in late 2020.[7]”
Reviewer: Why solely gonorrhea and not also chlamydia and syphilis. See comments before. How is chlamydia and gonorrhea tested, separately tested and not together in a PCR test?

**Response: We revised this sentence accordingly to include the changes in trends for chlamydia, gonorrhea, and syphilis: “Following the CDC’s guidance, syphilis and gonorrhea were made a top priority by many health departments. This might explain the significant decline in chlamydia tests, which was potentially due to the limited resources, and the increase in gonorrhea tests for all patients and the increase in syphilis tests for certain populations in late 2020, compared to their testing rates in the same period in 2019.”**

**While we recognize that there exist differences in the chlamydia and gonorrhea testing rates, the database does not provide insights specifically into practice pattern differences in the ordering of tests. Primary care providers’ data are documented from each practice’s electronic health records but does not provide information regarding the process regarding how tests are ordered. We have added some language into the limitations section of the Discussion.**

“Another potential explanation for the increase in gonorrhea tests in late 2020 is that the stay-at home order ended in many states after June 2020.[30]”
Reviewer: Same question why gonorrhea only and not the other STI tests?

**Response: The testing rates for all STIs rebounded in June 2020 after a sudden drop between March and May 2020 when most of the states were under stay-at-home order. Among the all four STIs we were looking at, gonorrhea was the only one that reached a higher annual testing rate in 2020 than that in 2019. This could be attributed to a combined effect of reopening and prioritized resource allocation particularly in primary care settings. We updated the sentence to highlight this:**

**“The fact that the stay-at home order ended in many states since May 2020 may also have contributed to the testing rate increase later in the year.[7] This enabled primary care practices to resume services and perhaps increase STI testing due to increased demand post initial COVID-19 phase.[36] Although all STIs experienced increases in testing rates from April to June 2020 and remained in a relatively stable level for the rest of the year, gonorrhea was the only one that had a higher annual testing rate for all patients in 2020 than that in 2019. This could be related to a combined impact of the resource allocation priority and reopening. This also indicates that in primary care practices within the PRIME Registry network, gonorrhea might have been given the top priority over other STIs during that time.”**

“Black or African American patients were seen at a higher risk of having STIs and related conditions than other racial/ethnic groups.[32]”
Reviewer: Hispanic patients are not seen as at higher risk?

**Response: We thank the reviewer for highlighting this. We revised it as: “Black or African American patients and Hispanic patients were seen at a higher risk of having STIs and related conditions than White patients.**”

“Another potential limitation is that tests were identified using procedure codes; therefore, tests that were documented in free-text fields were not captured in this study.”
Reviewer: Can you quantify how many and the impact.

**Response: We thank the reviewer for raising this point. In our study, we mainly extracted procedure codes from procedure tables, where less than 1% of the records have missing codes. If the procedure codes in procedure tables were missing for a patient, we searched for valid procedure codes in the lab test result tables. Around 70% of the records in the lab test result tables have missing codes but with descriptions in free text. However, many of those free-text descriptions only contain words such as “chart accessed” or “reviewed medications”, making it difficult to accurately identify the tests they refer to. Without sufficient information and an understanding of why a code is missing, it is hard to assess its impact on our analysis.**
