# Supplementary material for: Primary care screening for sexually transmitted infections in the United States from 2019 to 2021
Source: PLoS One. 2025 Jun 2;20(6):e0325097. doi: 10.1371/journal.pone.0325097 (PMC12129226; doi:10.1371/journal.pone.0325097)
Supplement: Attachment — Submitted filename: Response letter STI Y2_2025_2.docx [file pone.0325097.s007.docx]

Journal Requirements:

Please review your reference list to ensure that it is complete and correct. If you have cited papers that have been retracted, please include the rationale for doing so in the manuscript text, or remove these references and replace them with relevant current references. Any changes to the reference list should be mentioned in the rebuttal letter that accompanies your revised manuscript. If you need to cite a retracted article, indicate the article’s retracted status in the References list and also include a citation and full reference for the retraction notice.
**Response: We’ve reviewed and updated the references in the revised manuscript. Specifically, we replaced the original references 2, 18-20, 27, and 29 with new ones as their web links are no longer valid or might be invalid soon. All references in the revised manuscript are now complete and correct.**

**Comments to the Author**

Reviewer #3: This article presents an interesting study on the changes in monthly and annual testing rates for chlamydia, gonorrhea, syphilis, and HIV in primary care practices in the US during the COVId-19 pandemic compared to pre-pandemic levels.
The study shows a significant drop in STIs testing in the primary healthcare setting, particularly during stay-at-home orders, followed by a recovery after the relaxation of those orders, although testing rates did not return to 2019 levels. Interestingly, gonorrhea testing rates increased in 2020 compared to 2019, and females and non-Hispanic Black or African Americans patients exhibited higher annual testing rates.
The manuscript is generally well written and clearly and logically presented. However, in my view, there are some areas that require review and revision before it can be accepted for publication. Please find my comments below:

Abstract
The authors should specify in the abstract the country/region where the study was conducted.

**Response: We added this information to the Abstract: “This study examined trends in STI testing and positivity before and during the COVID-19 pandemic in the primary care setting in the United States.”**

The results section of the abstract should include some numerical data, rather that only stating increases or decreases.
In the last sentence of the results section, “Test positivity during the pandemic was elevated for both chlamydia and gonorrhea”, the term ‘elevated’ is unclear. what does mean “elevated”? The authors should provide specific numbers and clarify the comparison baseline.

**Response: We thank the reviewer for highlighting this. We modified the result section as:**

**“We observed a substantial decline in testing rates for STIs from January-April 2020 (53% for chlamydia, 46% for gonorrhea, 36% for syphilis, 42% for HIV), followed by a rapid increase in May-June 2020 (64% for chlamydia, 65% for gonorrhea, 32% for syphilis, 48% for HIV). Chlamydia testing rates decreased from 2019 to 2020 (2,355 vs 3,592, per 100,000), while gonorrhea testing rates increased during this time (2,207 vs 2,129, per 100,000). STI testing rates for females and non-Hispanic Black or African American patients were higher than for other groups during the pandemic. Test positivity was higher in Q2-Q4 in 2020 than in 2019 for both chlamydia (1.6% vs 1.4%) and gonorrhea (0.5% vs 0.3%).”**

Introduction
(No specific comments)

Methods
The secondary outcome was the test positivity for chlamydia and gonorrhea. Why weren’t test positivity rates for syphilis and HIV included as secondary outcomes?

**Response: We thank the reviewer for raising this concern.**

**Unlike chlamydia and gonorrhea, it is hard to confirm a “new” incidence for syphilis and HIV with our data. A presumptive diagnosis of syphilis requires use of two laboratory serologic tests, so one positive test result of syphilis doesn’t necessarily mean a new infection. For HIV, since it is a lifetime condition and some patients may have been diagnosed outside the PRIME Registry network, we are unable to determine a new incident HIV simply based on positive test results documented in our data. These issues are related to data integrity and interoperability, underscoring challenges of EHR-based studies. Given the complexity and uncertainty of calculating the positivity rates for syphilis and HIV, we didn’t include positivity rates for these two conditions in the manuscript.**

In the first line of the Patient Characteristics section, eliminate the extra ‘r’ in ‘sexr’.

**Response: We’ve changed it to “gender”.**

Results
(No specific comments)

Discussion
Overall, I found the discussion section too brief. It would benefit from, a comparison of these results with studies conducted in other setting or in other countries/regions. This would enable a deeper understanding of discussion of the hypotheses regarding the increase in gonorrhea testing rates compared to other STIs, as well as the higher testing rates among females and non-Hispanic Black or African American patients.

**Response: We thank the reviewer for highlighting this. We revised the third paragraph in Discuss by adding a comparison with other studies:**

**“Notably, some US-based studies reported a decrease in gonorrhea testing volumes in 2020 compared to 2019 [33, 34].** **This might reflect variations in resource allocation patterns across different clinical settings during the pandemic. This might also indicate an improvement in gonorrhea test documentation within structured EHR systems in PRIME practices, which could result in higher observed gonorrhea testing rates.”**

**We also revised the 4th paragraph in Discussion:**

**“Our findings that females and non-Hispanic Black or African American patients having higher annual STI testing rates during our study period are consistent with the results of a few studies.[33-37] This may be explained by several phenomena. The high rate amongst females could be due to the U.S. Preventive Services Task Force (USPSTF) recommendations for STI screening for sexually active young women or due to pregnancy screening.[38] However, there is insufficient evidence in our study to suggest if STI testing is screening or diagnosis. Studies based on EHR, surveys, and claims data also found that Black or African American patients were more likely to receive STI tests compared to Whites.[35-37, 39] This is potentially related to the finding that this population were seen at a higher risk of having STIs and related conditions than White patients,[2, 40] which might have led to a risk-based prioritization in providing STI testing services.[39] Further, research within a U.S. hospital system found that despite a reduction in overall STI testing counts during the early pandemic, females and non-Hispanic Black or African American patients had increased testing proportions compared to males and Whites.[33] These findings, together with ours, may reflect that the clinical settings prioritized certain populations for STI testing in response to the public health emergency.[5]”**

The stated limitation about symptom presentation at the time of testing, which prevents distinguishing between tests conducted for screening versus diagnosis, is critical. While this is an important limitation, the discussion could be enriched by incorporating findings from other studies. For instance, the authors could address how the higher symptom presentation for gonorrhea compared to chlamydia, as well as the potential role of pregnancy screening (as mentioned by the authors), might have influenced the results.

**Response: We added the following sentences to the study limitation to discuss the role of symptom presentation and pregnancy screening:**

**“Furthermore, we do not know symptom presentation at the time of testing from the procedure codes; therefore, we are unable to discern tests for screening versus diagnosis or investigate how symptom presentation influenced the relationship between patient-level characteristics and testing rates. Additionally, given that a large proportion of chlamydia and gonorrhea cases are asymptomatic,[41-43] routine screening for STIs is essential for early identification and treatment.[41, 43] We are unable to examine the proportion of routine screening, though. We are also unable to access whether clinics prioritized tests to symptomatic patients due to limited resources during the COVID.[33, 34]”**

**“Pregnancy-related data were not collected in this study. The role of pregnancy-related screening in testing rates and the adherence to USPSTF and CDC’s recommendation,[38, 44, 45] therefore, is unknown.”**

Conclusion
The authors should mention the country/region where the study has been conducted.

**Response: We modified the conclusion to highlight the place where the study was conducted: “This study investigated the monthly and annual testing rates for chlamydia, gonorrhea, syphilis, and HIV in the U.S. primary care practices.” We also modified the title and abstract accordingly.**

Reviewer #4: Thank you for the opportunity to review this paper, that has been previously reviewed and commented on. I have checked the previous reviewers’ comments, which the authors seem to have responded to appropriately. The only outstanding concern I have which is shared by the other two reviewers. In New Zealand the PCR test we use to test for Chlamydia and Gonorrhea tests for both infections at the same time. The health provider is not able to choose only one. So you will never receive test results for Chlamydia only or Gonorrhea only, it will always contain two results. I'm not sure if it’s the same in the US, but your previous reviewers are questioning why you would have more results in your database for Gonorrhea than you would for Chlamydia when both are tested for at the same time (and both results reported). I don't think you have answered this satisfactorily. Check with the lab whether this is the case and if it is you will need to try and find out why you have more results for one test than the other. If the lab tests them separately then make this explicit so that overseas readers like me are not left wondering about the discrepancy.

**Response: Thank you for bringing this to our attention.**

**While chlamydia and gonorrhea are often tested together in primary care in the United States, the data source used for this study documented these tests separately. In this study, over 60% of the chlamydia tests were documented using chlamydia-specific CPT codes 87490 and 87491, and over 85% of the gonorrhea tests were documented using gonorrhea-specific CPT codes 87590 and 87591. Additionally, 53% of the chlamydia tests and 92% of the gonorrhea tests can be matched together based on the test dates and patient information, suggesting that these tests were ordered together for the same patients. We were unable to map the rest 47% of the chlamydia tests to any gonorrhea tests, however. Further, in our data, 35% of the practices documented chlamydia tests but didn’t record any gonorrhea tests during the same period. These resulted in a large difference in counts between the two tests, as shown in Table 1.**

**The discrepancy in counts could be likely related to practice-level factors. These practices might not have submitted gonorrhea test files to our database, or the files they submitted were filtered out by the system due to issues like data quality. Additionally, gonorrhea-related information might be recorded in unstructured formats or in notes, which are currently not accessible to us.**

**We’ve added a footnote to Table 1 to outline these potential reasons: “Chlamydia and gonorrhea tests are often ordered together in primary care. However, in our data, a few practices documented chlamydia tests in the EHR but didn’t document any gonorrhea tests throughout the entire study period, resulting in a difference in total counts between the two tests. The absence of gonorrhea test documents from these practices could be due to variations in EHR data quality and integrity, which limits our access to them.”**

Some further suggestions to aid with clarity of the manuscript:
1. when you talk about race and ethnicity I think it would be better to group them together as "race/ethnicity". At the moment it sounds like you have two separate measures, one for race and one for ethnicity when I think you only have one data point, is that correct?

**Response: Thanks for pointing this out. We’ve changed “race and ethnicity” to “race/ethnicity” throughout the manuscript.**

2. Table one - you could remove most of the repeated headings for each column and put them in the title instead. So take out: "patients who had" and "2019-2021" and change the table heading to something like: Characteristics of patients who had STI tests, American Family Cohort, 2019-2021. The column with no testing could be "No record of STI testing".

**Response: Thank you. We’ve updated Table 1 accordingly.**

3. Could you make it explicit in the title of the paper, or the abstract that the study was carried out in the US?

**Response: To highlight the country where the study was carried out, we changed the title to: “Primary care usage patterns associated with sexually transmitted infections in the United States during COVID-19.” We also added this to the Abstract: “This study examined trends in STI testing and positivity before and during the COVID-19 pandemic in the primary care setting in the United States.”**

4. I'm not sure whether I agree with Primary Care as a locus of monitoring mentioned in your conclusion. Its focus is really to screen, diagnose and treat. Its more that primary care data provides an opportunity for monitoring.

**Response: We agree with the reviewer’s point and have modified the abstract and conclusion accordingly: “This study emphasizes the role of primary care EHR data in monitoring and an opportunity for closer collaboration with public health agencies.”**
