# Supplementary material for: Primary care screening for sexually transmitted infections in the United States from 2019 to 2021
Source: PLoS One. 2025 Jun 2;20(6):e0325097. doi: 10.1371/journal.pone.0325097 (PMC12129226; doi:10.1371/journal.pone.0325097)
Supplement: Attachment — Submitted filename: Response letter STI Y2_2025_3.docx [file pone.0325097.s008.docx]

Suggestions for the title and abstract:
1. The short title of the paper is clearer than the full title.
Full Title: Primary care usage patterns associated with sexually transmitted infections in the United States during COVID-19
Short Title: STI screening in U.S. primary care during COVID-19

Please rewrite the full title to more clearly reflect the research question’s exposure variable. The full title suggests that the primary exposure variable is primary care usage patterns, such as number of visits per year, such as comparing patients who made 2+ visits per year with 1 visit per year. It seems from the results that your exposure variable is the year 2020 versus the year 2019. With the three years 2019-21, it seems that you have three periods: before covid-19, covid-19 before the vaccine, and covid-19 after vaccine. Either use 2 periods (before and after covid) or 3 periods (before, pre-vaccine, post-vaccine).

**Response: We thank the Editor for raising this. Our analysis covers a three-year period from 2019 to 2021 in the US. Since this period was across the onset of COVID-19 and the stay-at-home order in March 2020, we would like to investigate how COVID-19 impacts the annual and monthly patterns of STI testing rates, plus the test positivity. While we are cognizant that there are three distinct periods, our analysis primarily wishes to explore these trends across the entire time frame with some attention to each of the three periods (before COVID, pre-vaccine during COVID, and post-vaccine during COVID).**

**We’ve updated the full title as “Primary care screening for sexually transmitted infections in the United States from 2019 to 2021”.**

2. Sample sizes go in the methods section: n=180558 patients tested for STIs out of 4410609 patients seen at 753 primary care practices over 3 years.

**Response: We moved the sample size description to the Methods: “753 primary care practices and 4,410,609 patients were included, with 180,558 having one or more STI tests.”**

3. You have 3 years of data: 2019, 2020, and 2021, but the entire results section of the abstract only refers to the first two years. Please write the abstract so that the methods section says specifically 3 years and so that in results section you are referring more clearly to the data. I don’t see any references at all to any data from January 1 2021 to December 31 2021. I might expect each result to have 3 numbers, one for each of the years of the data. Please also refer to the years in the title of the paper.

**Response: We thank the Editor for highlighting this.**

**We rewrote the Results section by adding data in 2021: “Testing rates per 100,000 decreased from 2019 to 2021 for chlamydia (3,592 vs 2,355 vs 2,181) while increased for gonorrhea in 2020 (2,129 vs 2,207 vs 2,057). STI testing rates from 2019 to 2021 for females and non-Hispanic Black or African American patients were higher than other groups.”**

4. The title refers to “during covid-19” and in the results section of the abstract you refer to three different time increments: months, quarters, and years. Statistically, using multiple time periods is a concern because making multiple comparisons can cause false significance.

More specific to the subject matter, the time divisions do not correspond naturally to the subject matter of the covid-19 pandemic. For example, you note that there is a decrease in January to April 2020 followed by an increase in May-June, but the rationale for this time division is not clear because January and February 2020 and the first week or two of March are not “during COVID-19” in any meaningful sense. We would not expect that time period to have different STI testing patterns. Please use a time division that corresponds to start of pandemic and optionally a second division after vaccine availability.

**Response: We revised the time division for STI testing rates at early pandemic: “We observed a substantial decline in testing rates for STIs from March-April 2020 (31% for chlamydia, 30% for gonorrhea, 23% for syphilis, 24% for HIV)”. We also rewrote the test positivity results in annual trend for consistency: “An increase in test positivity from 2019 to 2021 was observed for gonorrhea (0.4% vs 0.4% vs 0.5%) but no significant change for chlamydia (1.5% vs 1.6% vs 1.5%).”**

**We also updated the title and Background section accordingly to highlight the study period: “This study examined trends in STI testing and positivity from 2019 to 2021 in primary care in the United States.”**

5. Please give a couple of words describing the PRIME registry for readers who have never heard of it before.

**Response: We revised the first sentence in Methods: “This is a retrospective study using the PRIME Registry, a national primary care EHR registry, from January 1, 2019-December 31, 2021.”**

6. Maintain parallelism. In the abstract results section, you give the years 2019 and 2020 in that order, but in parentheses you list the number of people tested for chlamydia and gonorrhea in 2020 followed by 2019. As noted above, though, I think you should have well-defined and meaningful time periods with respect to covid-19.

**Response: We thank the Editor for highlighting this. We’ve revised this and presented the testing rates in an order of 2019, 2020, 2021 for chlamydia and gonorrhea. We also included the drop in % for all four STIs from March to April 2020.**

7. The methods section must specify any statistical tests that you used, and I don’t see any statistical analysis methods referred to. You calculated the age-adjusted testing rates, but you do not refer to an analysis method to evaluate whether one rate differs from another.

**Response: We added at the end of Methods: “Chi-square tests and 95% confidence intervals were used for comparison.”**

Analysis:
1. Age adjustment: please be specific about which US Census data was used for age adjustment. I see from figure legends that you adjusted by US population. Please specify US country-level age adjustment or a less clunky term to be clear. Otherwise I would have thought each clinic had a catchment area and you adjusted by the county age distribution for each location or similar.

**Response: We’ve added the source data we used for age adjustment: “Direct age adjustment was based on using the reference U.S. age distribution per the U.S. Census 2020 data for the study period. This is based on Annual Estimates of the Resident Population by Sex, Age, Race, and Hispanic Origin for the United States: April 1, 2020 to July 1, 2021.”**

2. Keyfitz formula is age-adjusted rate divided by square root of the number of events. It is not complicated, please actually state that instead of making the reader look it up. You do not show the error bars on your figures, so it is hard to tell what is different.

It may be more natural to at least attempt a basic time series analysis. Perhaps you will not see anything, but sometimes you can see a pattern in these pre vs during covid data. (We did this in our publications on pediatric GI diseases.) I see a decrease in both Januaries without covid in 2019 and 2020, and maybe there is a seasonal component to STI testing.

**Response: We added the formula for calculating 95% confidence intervals of age-adjusted rates:**

**“The 95% confidence interval (CI) was calculated based on the standard errors generated using the Keyfitz formula:**

$$95\% CI= \pm1.96\times\frac{R}{\sqrt{N}}$$

**where R is the adjusted rate for STI testing, N is the number of tests.”**

**We agree with the Editor that time series analysis would allow us to further explore seasonal patterns of STI tests in primary care, providing insights into underlying potential drivers. Testing behaviors can be influenced by multiple factors. Some patients may come for tests due to symptoms and seek an STI diagnosis, while others may be asymptomatic but at increased risk. Certain groups of patients may also come for annual routine screening, like women aged 15 to 24 who are sexually active. Seasonal patterns of tests, therefore, can be driven by various motivations. However, due to the limitation in our data source, we are unable to differentiate a routine screening from a diagnostic test or identify the motivations for screening. Further expounding upon potential limitations to draw inference about this variation is based on not knowing lifestyle and risky sexual behaviors as being drivers of this variation across time since these are not captured in the PRIME Registry in any reliable form. Lifestyle and sexual behaviors (or risk taking) could influence screening motivation or the patient’s suspicion that they may have an STI thereby driving up screening in this population. Due to these limitations, it is tenuous to link seasonal patterns of tests to seasonal patterns of infections. We plan to include this analysis in future studies using external linkage datasets with increasing patient details which can help explain these variations in testing and test positivity.**

3. Figures are nice, but I don’t think either figure 1 or 2 is the optimal data display. Consider modifying figure 1 so that it separates the diseases, so make figure 1 into 4 plots. That will give you space to put the error bars on each point or some other means of communicating to the reader what is different.  I suggest further putting dotted line markers at time points that are meaningful for covid pandemic. If you want 2 time periods put one somewhere in March 2020 and if you want 3, put a second line at a meaningful time with respect to vaccine availability such as April 2021.

**Response: We’ve revised Fig 1 accordingly. Testing rates for each STI were plotted separately with error bars. A dotted line was added to indicate the beginning of stay-at-home order in the U.S. in March 2020.**

4. You have an enormous amount of supplementary information. Given PLOS ONE has pretty minimal manuscript requirements with no word limits or figure limits, much of the information can be incorporated into the paper and then the supplementary information will be minimized or eliminated. Readers do not in practice read as much of the supplementary information as much as they read the paper. When faculty distribute published papers to their students, the supplementary information is not included.
     a. Information in S2 file seems pertinent to your methods section. Please include more of that information in paper body.

**Response: S1 file and S2 file were incorporated into the main text.**

    b. S2 table is better as a map. R will produce a US map with numbers in the states. Here are some options: <https://jtr13.github.io/cc19/different-ways-of-plotting-u-s-map-in-r.html>
I think other software tools will do this as well.

**Response: We plotted the data from S2 Table as a map and renamed it as S1 Fig.**

c. S3 table would be good to include as a table within the paper itself as opposed to supplementary.  Another way to arrange the data that may be clearer is by disease rather than by year, so that the columns are 2019, 2020, and 2021, allowing comparison of chlamydia across all the March and April, for example.

**Response: We re-arranged the data in S3 Table by STI and moved it to the main text (Table 2).**

    d. Figure 4 and S1 and S2 figures overlap a lot. I suggest just making figure 4 and 5, where figure 4 is all diseases by gender and figure 5 is all diseases by race/ethnicity so you can take out the corresponding supplementary figures.

**Response: We combined Fig 4 and S1 Fig into a new Fig 4 and Fig 5.**
